# Bell’s Palsy: Description, Diagnosis, and Current Management

**DOI:** 10.7759/cureus.77656

**Published:** 2025-01-19

**Authors:** Seth Gardner, Lexi Garber, John Grossi

**Affiliations:** 1 Department of Anatomy, Lake Erie College of Osteopathic Medicine, Bradenton, USA

**Keywords:** bell’s palsy, facial nerve, idiopathic facial paralysis, seventh nerve palsy, stroke

## Abstract

Bell’s palsy, also known as idiopathic facial paralysis, is a neuropathy that affects the seventh cranial nerve, the facial. Its presentation is unmistakable by a trained clinician. It usually presents with unilateral facial weakness, reduced forehead wrinkling, nasolabial fold flattening, drooping of the corner of the mouth, and drooling. Its comorbidities are thought to include diabetes, obesity, and pregnancy. It is a diagnosis of exclusion with treatments ranging from corticosteroids to antivirals, to a combination of both corticosteroids and antivirals, to acupuncture, physical therapy, and chiropractic. Most patients will recover fully within six months regardless of the treatment.

## Introduction and background

Bell's palsy is a widely studied pathology and is quite common to diagnose in clinics; however, the etiology is unclear in the majority of cases. This literature review will discuss the risk factors, pathogenesis, and common clinical presentations of Bell's palsy. Additionally, this literature review will address how to diagnose Bell's palsy in the clinic and different treatment plans. There is a vast majority of research that is still evolving regarding this topic, and by doing further exploration, this review article can help medical providers better recognize and manage Bell's palsy in the clinic. Bell's palsy is the most common peripheral paralysis of the seventh cranial nerve [1]. Its onset is reported to be sudden and unilateral [1]. Bell’s palsy, also called cranial nerve VII or facial nerve palsy, is a sudden-onset facial weakness with several potential triggers [2]. It is an acute paralysis of the muscles innervated by the facial nerve [3]. By collecting a thorough patient history, physical examination, and diagnostic studies, such as labs and imaging, it is a diagnosis of exclusion [4]. Providers may mistakenly generalize a diagnosis of Bell’s palsy in the setting of a potential underlying etiology, such as Ramsay-Hunt or Lyme disease [1]. In the 19th century, Sir Charles Bell described Bell’s palsy as an acute, ipsilateral facial paralysis [4]. He described the facial nerve as “the respiratory nerve of the face. It controlled the motions of the face which relate to respiration" [5]. Bell described it as an acute-onset, idiopathic facial paralysis due to a dysfunction anywhere along the peripheral portion of the facial nerve [6]. Initially, all cases of facial nerve paralysis were called Bell's palsy; however, after the causes of the disease were discovered, only the idiopathic cases were called Bell's palsy [7]. Peripheral facial nerve palsy refers to a lower motor neuron lesion of the facial nerve and can occur because of various medical conditions such as infection, cholesteatoma, trauma, malignancy, autoimmune issues, and pregnancy [8].

The facial nerve is derived from the second branchial arch [9]. This arch also gives rise to the muscles of the face, the occipitofrontalis muscle, the platysma, the stylohyoid muscle, the posterior belly of the digastric muscle, the stapedius muscle, and the auricular muscles [9]. All of these muscles are innervated by the facial nerve [9]. This nerve also controls salivary and lacrimal glands [1]. The facial nerve has an intracranial, infratemporal, and extratemporal course as its branches [10]. It also conveys parasympathetic function to the lacrimal and salivary glands, except the parotid, as well as taste from the anterior two-thirds of the tongue [10]. The facial nerve exits the brain stem from its ventrolateral surface at the cerebellopontine angle [10]. It consists of two parts: a proper facial nerve and an intermediate nerve [10]. The proper facial nerve contains only a motor component and a small somatic afferent component [10]. However, the intermediate nerve carries sensory and parasympathetic visceromotor components [10]. Diagnosing Bell’s palsy can be quite difficult because there are many differential diagnoses that can present similarly, including trauma, neoplasm, and infection [11]. Bell’s palsy is typically a self-limiting disorder with a favorable prognosis; however, its abrupt onset, rapid progression, and dramatic presentation can be frightening, especially when it occurs in a child [12]. There are more favorable outcomes of facial nerve palsy in the pediatric population as compared to adults [13]. It has been studied that younger patients with facial nerve palsy have a complete recovery within six months [13].

## Review

Methods

**Figure 1:**
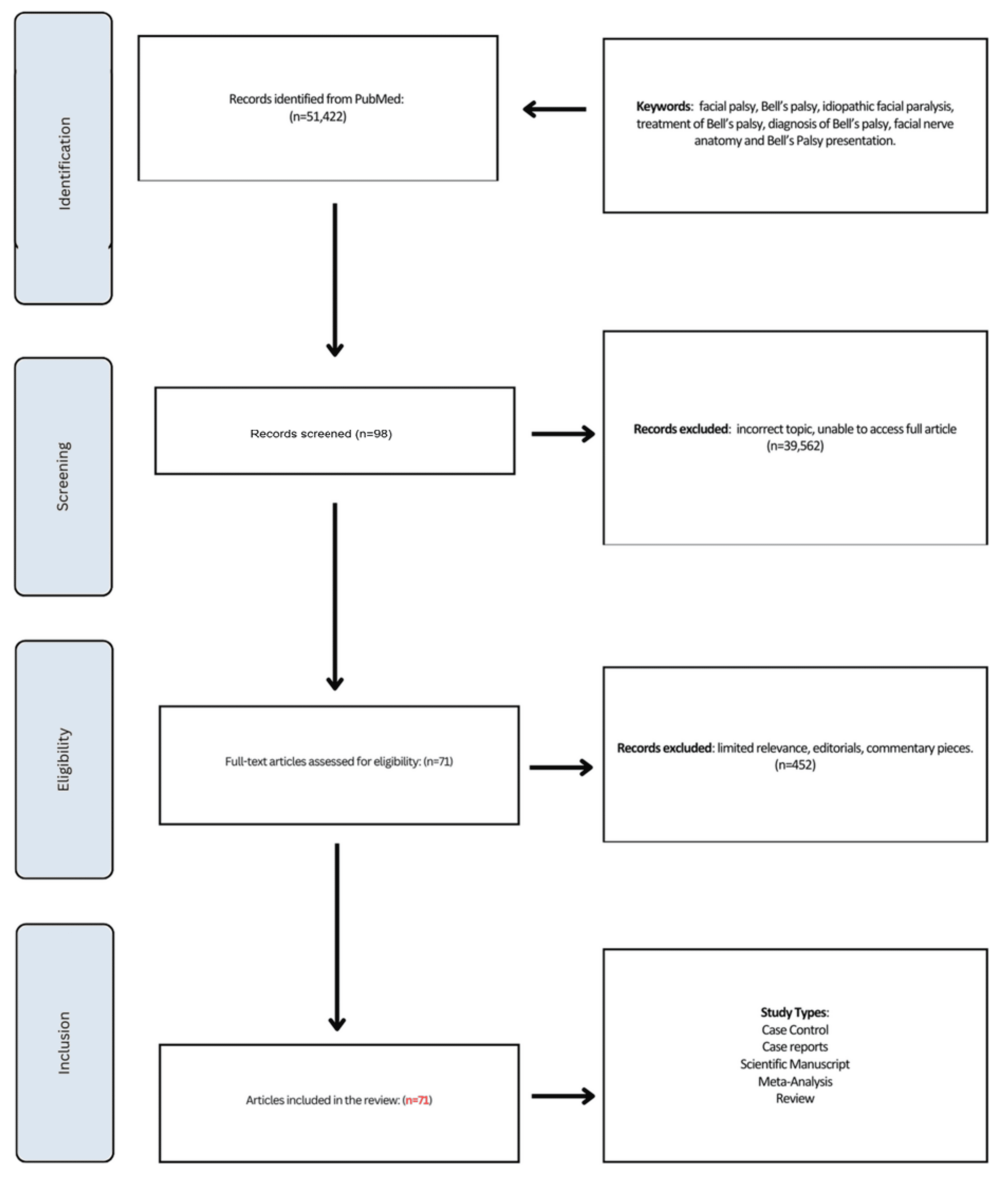
Preferred Reporting Items for Systematic reviews and Meta-Analyses (PRISMA) diagram illustrating data collection.

In this review, free full-text articles were searched on PubMed from January 1996 to January 2024 using the following search terms: facial palsy, Bell’s palsy, idiopathic facial paralysis, treatment of Bell’s palsy, diagnosis of Bell’s palsy, facial nerve anatomy, and Bell’s palsy presentation. A Preferred Reporting Items for Systematic reviews and Meta-Analyses (PRISMA) diagram is shown in Figure 1 to illustrate our data collection.

Epidemiology and risk factors

Some research shows that the annual incidence of Bell’s palsy is 11.5-53.3 per 100,000 persons [4]. Another author reports that the global estimated incidence of Bell's palsy ranges from 11 to 40 cases per 100,000 person-years in adults and 11.5 to 30 per 100,000 persons in children [14]. With a lifetime risk of one in 60, the median onset of Bell’s palsy is roughly 40 years of age; however, it can be seen in all age groups [4]. Epidemiologic studies show that facial nerve palsy has a higher incidence in people aged between 15 and 40 years, with the peak being closer to 40 [15]. Other studies have suggested that the incidence rate is highest among young and middle-aged adults [16].

Some of the major risk factors for developing Bell’s palsy include pregnancy, preeclampsia, obesity, and hypertension [1]. Other risk factors for Bell’s palsy include obesity and upper respiratory diseases [17]. One study found a higher incidence rate of Bell’s palsy among women than men in their study population [12]. It seems there is a slight female preponderance, and one author found that Bell’s palsy is diagnosed in the spring and fall more frequently than any other time of the year [18,19].

Bell’s palsy disproportionately affects pregnant women; diabetics; those who experience upper respiratory tract infections, such as influenza and rhinovirus; and those who have undergone tooth root extraction [19]. It was also found that hypertension measured during the initial presentation during a hospital visit was significantly associated with worse outcomes, after given Bell’s palsy diagnosis [20]. The American Academy of Otolaryngology-Head and Neck Surgery clinical practice guideline for Bell’s palsy reiterates that patients who are pregnant or have severe preeclampsia, obesity, chronic hypertension, diabetes, and upper respiratory ailments are at a higher risk for Bell’s palsy [21]. Another epidemiologic study in Italy showed that advanced age is a significant risk factor for Bell’s palsy, and there is even a linear trend that exists as a correlation between older age and risk for developing Bell’s palsy [22]. Although there has been an effort to assess the relationship between socioeconomic factors, such as income and location of residence, and the incidence of Bell’s palsy, no associations have been made [23]. Interestingly, in Korea, the findings are unique given that male sex and residence in a location other than the capital and metropolitan cities were significant risk factors for Bell’s palsy [23]. It has also been reported that adverse weather conditions are considered to increase the likelihood of developing Bell’s palsy [24].

Several comorbidities associated with Bell’s palsy have been reported, such as high blood pressure, diabetes, hypercholesterolemia, and other dyslipidemias [25]. Interestingly, it has been suggested that statin drugs and their neurotoxic effects could increase the risk of Bell’s palsy [26]. Statins inhibit the synthesis of ubiquinone, which is essential for the mitochondrial respiratory chain, and in doing so, it creates a depletion of coenzyme Q10, and the health of the nerve is affected due to the disruption of neural growth and function [27]. A previous health claim cohort study proposed that there is a relationship between statin use for six months and increased risk of Bell’s palsy [28]. However, the adverse effects of statins on Bell’s palsy might be insignificant and are not well defined [26]. Some clinical studies have reported the neuroprotective and anti-inflammatory effects of statins, indicating that these medications might have a protective role against peripheral neuropathy [29]. Therefore, the research does not agree on whether statins can be harmful or beneficial to Bell’s palsy and other neuropathies. The adverse effect of statins on neuropathy was as low as one person/14,000 person-years of treatment [30].

There are at least two microorganisms that are known to cause Bell’s palsy [31]. These infectious pathogens include *Borrelia burgdorferi*, which causes Lyme disease, and varicella zoster virus, which causes Ramsay-Hunt syndrome [31]. However, there are conflicting results concerning the role of *B. burgdorferi* in the occurrence of Bell’s palsy [31]. Some studies found that there is an increased prevalence of *Borrelia* antibodies among patients with Bell’s palsy [32]. However, there is still controversy on whether this correlation exists. Increasing evidence suggests that Bell’s palsy is caused by a reactivation of latent herpes viruses, either herpes simplex or herpes zoster [32]. There are many reports on the association between facial paralysis and viral infections, for example, varicella zoster, which causes Ramsay-Hunt syndrome [33]. Furthermore, with the age of the population increasing, there is an increased prevalence of immunocompromised patients, which can ultimately increase the reactivation of latent herpes simplex virus infections and, thus, result in a higher risk for Bell’s palsy [23]. Bell's palsy remains idiopathic, but a proportion of cases may be caused by the reactivation of herpes viruses from the geniculate ganglion of the facial nerve [34]. Interestingly, facial palsy as a presentation of Eagle’s syndrome is extremely rare until Rosales et al. made the first case report of a 40-year-old male patient who presented at the emergency department with facial palsy and symptoms of Eagle’s syndrome [35].

Pathogenesis

Bell's palsy is idiopathic in nature [1]. The etiology of Bell’s palsy involves inflammation and ischemia of cranial nerve VII [1]. This inflammation and ischemia are due to the compression of the nerve as it travels through the geniculate ganglion inside the facial canal [1]. Other associated etiologies of facial paralysis not listed previously can include congenital, traumatic, metabolic, neoplastic, toxic, and vascular causes [36]. It is well accepted that most cases of Bell palsy are related to virus-induced inflammation of the facial nerve; however, there are a few cases that are related to acute otitis media [37]. Also, a cell-mediated autoimmune response against myelin has been linked with Bell’s palsy [38]. Blum reported that intracranial mechanical stress can affect cranial bones and meninges, which can lead to entrapment neuropathies. This is especially true of the facial nerve because of its relationship with the arachnoid and dura mater [39]. Another thought is that the thickening of the vessels and edema surrounding the nerve could compress the facial nerve itself [40]. Therefore, patients with uncontrolled hypertension have an increased risk of hemorrhage into the facial canal and necrosis due to the thickening and edema that occurs, which can ultimately compress the facial nerve [23].

Bell’s palsy can also occur due to either congenital or developmental causes [36]. Congenital palsy occurs due to perinatal trauma [6]. Developmental palsy occurs due to errors in development, for example, aplasia or hypoplasia of either the cranial nerve nuclei or the facial nerve itself or nuclear agenesis [41]. Although the exact cause of Bell’s palsy is not known, it is thought that the recurrence of a dormant viral infection can cause the facial nerve to swell and become inflamed [42].

Clinical presentation

Bell’s palsy presents acutely; therefore, emergency medicine clinicians are more likely to see these patients than medicine subspecialists [43]. The palsy is often sudden in onset and evolves rapidly, with maximal facial weakness developing within two days. The classic features of Bell’s palsy include forehead wrinkling, ptosis of the eyebrow, drooping of corners of the mouth, flattening of the nasolabial fold, and incomplete closure of eyelids [44]. The sparing of the forehead and eyebrow muscles is attributed to the bilateral innervation of the upper third of the face and contra-lateral innervation of the lower two-thirds [45]. Hyperacusis, decreased production of tears, and altered taste may also be noted [31]. Moreover, nasolabial folds may completely disappear, the forehead can unfurrow, and the corners of the mouth droop in affected patients [4]. Symptoms can range from mild to severe facial paralysis [46]. Symptoms can also include trouble blinking, impeded speech, and incomplete smiling [46]. Facial motor weakness may be accompanied by other symptoms such as dysgeusia, xerostomia, altered facial sensation, vestibular dysfunction, and pharyngeal paresthesia [32,47]. Additional symptoms of Bell's palsy can include pain surrounding or in the ear, oropharyngeal or facial numbness, hyperacusis, and altered taste on the anterior portion of the tongue [34]. These symptoms and signs occur because the facial nerve carries motor fibers to the stapedius muscle and supplies autonomic innervation to the lacrimal gland and submandibular gland, sensation to part of the ear, and taste to the anterior two-thirds of the tongue [48]. Most of the affected individuals think they have had a stroke or a serious brain lesion [31]. It is essential to also note if Bell’s palsy is unilateral or bilateral [36]. Unilateral facial nerve palsies are usually idiopathic or viral-related, whereas bilateral palsies are usually due to an underlying systemic pathology [36]. Although bilateral facial nerve palsies are not as common, these patients should still receive a thorough workup as an underlying etiology could be present [36]. Either way, most of these sequelae affect the individual both socially and psychologically, leading to a decline in their quality of life [49].

Diagnosis

There appears to be no diagnostic marker for Bell’s palsy. It remains a clinical diagnosis and an exclusion diagnosis, as implied by the idiopathic label [21]. The history and physical examination are generally sufficient to diagnose Bell’s palsy [4]. Imaging is usually unnecessary [50]. Bell’s palsy can be a difficult diagnosis to make, especially with acute stroke being a differential diagnosis, as both can present very similarly to each other [11]. Central nervous system pathologies, such as multiple sclerosis, and certain strokes or tumors can also cause facial nerve palsy [38]. When diagnosing Bell’s palsy, the auditory canal should be inspected for vesicular lesions to exclude herpes zoster [51]. Bell’s palsy is diagnosed when there is a sudden onset of impaired facial expression due to weakness of facial muscles and their nerve branches [52]. This results in dry eyes, the inability to close the eye or mouth and wink, and drooping eyebrows or the corners of the mouth [52]. Numbness, ear pain, altered sense of taste, and hyperacusis can also occur [52].

Diagnostic imaging is not needed to establish the diagnosis of Bell’s palsy unless there are concerning features, such as parotitis, or signs of increased intracranial pressure, which could suggest a tumor instead [51]. Magnetic resonance imaging (MRI) is the imaging modality of choice to rule out other causes [53]. MRI can detect facial nerve inflammation and rule out a possible schwannoma or a hemangioma [53]. A common MRI finding is enhancement of the labyrinthine portion of the facial nerve, where the facial canal is narrowest [54]. The poor vascularity of this segment is predisposed to ischemia and venous congestion, which may cause the high-intensity enhancement pattern seen on MRI in Bell's palsy patients [54].

The main diagnostic challenge is to rule out whether the lesion is in the periphery and not in the central nervous system [11]. Developing a facial palsy is known to cause psychological distress [55]. There is a possibility of being misdiagnosed with a stroke, which can lead to unnecessary imaging and treatment, causing anxiety in patients and their families [55]. Most grading systems of Bell’s palsy rely on the evaluation of symmetry, the degree of voluntary excursion of the facial muscles, and the degree of synkinesis [56]. The House-Brackmann Scale is a commonly used grading system to diagnose Bell’s palsy, which analyzes the degree of facial nerve dysfunction [57]. The scale ranges from I (normal) to VI (total paralysis) in patients suffering from Bell’s palsy [57]. The House-Brackmann Facial Nerve Grading System is used to describe the degree of facial nerve weakness [1]. Additionally, nerve conduction studies and electromyography may help determine outcomes in patients with severe Bell palsy [53]. The outcome of Bell's palsy depends on the severity of facial nerve degeneration [20]. Age, diabetes mellitus, hypertension, initial House-Brackman grade, and extent of facial nerve degeneration as recorded by nerve conduction studies affect the clinical outcome of Bell's palsy at three months after its onset [20]. The nerve conduction study is a reliable tool to assess the degeneration of nerve fibers and prognosis overall when performed 72 hours after onset [44]. This is useful in the early detection of patients with poor prognostic indicators [44]. Furthermore, it is an electrodiagnostic test that quantifies the compound muscle action potential (CMAP) generated during nerve conduction [44]. In this test, the supramaximal stimuli are applied in front of the ear, near the stylomastoid foramen across a standard voltage, and the evoked muscle potential is measured as amplitude with representation in units of millivolts [44]. The strength of the amplitude is proportional to the number of muscle fibers present; hence, the degenerated motor nerve fiber reflects a reduced amplitude when compared with the normal side [44]. The CMAP generated in affected facial muscle is directly proportional to the number of affected nerve fibers that have lost their motor function. The paralyzed side of the face is compared with the normal side, and the amount of degenerated nerve fibers is quantified, allowing us to assess the severity of the palsy and the patient’s prognosis [44].

Conventional pharmacological treatment

The primary concern while treating Bell’s palsy patients is to find underlying issues that can be related to their diagnosis [58]. While 80%-90% of patients will recover fully, there are still those with residual deficits, such as difficulty with eye closure, asymmetric smile, eyebrow droop, or difficulty eating [43]. The treatment plan can vary based on external factors; however, most clinicians will prescribe steroids, such as prednisone or prednisolone [3]. Pharmacological management of Bell’s palsy without an apparent underlying cause includes the use of corticosteroids within the first 72 hours of onset of symptoms [59]. It is recommended that Bell’s palsy patients should be treated with oral corticosteroids, according to the American Academy of Neurology [4]. This will maximize the recovery of the facial nerve [4]. Another study also supports the use of steroids and found that the patients who used steroids, specifically prednisolone, had a better outcome than those who did not use them [60]. The response of Bell's palsy to steroid treatment also suggested that inflammation of the facial nerve plays a part in the pathogenesis of Bell's palsy [61]. There is also the option of using antiviral drugs to treat Bell’s palsy due to possible viral causes [62]. Some studies also propose that combined treatment of both steroids and antivirals can lead to better outcomes compared with using steroids alone [63]. Combinations of steroids and antiviral agents may have potential benefits, especially in patients with severe Bell’s palsy; therefore, treatment of Bell’s palsy should be based on individual patient characteristics [64]. There is limited to no current literature that supports the use of surgical decompression when treating Bell’s palsy, due to high costs and adverse outcomes [21]. Surgical decompression consists of deroofing the bony fallopian canal and incision of the facial nerve sheath as recommended by Coker [65]. Craniotomies carry risks, including seizures, deafness, leakage of CSF, and facial nerve injury [31].

Non-pharmacological treatment

Non-pharmacological treatments for Bell’s palsy include acupuncture, physical therapy, and hyperbaric oxygen therapy [66]. The effectiveness of acupuncture has been highlighted in multiple studies and is intriguing given its safety and low cost for patients [66]. Physical therapy, which includes biofeedback techniques, laser treatment, exercise, massage, and electrotherapy all aim to increase muscle function [67]. This is accomplished by decreased swelling, increased blood flow, and increased oxygen delivery to the affected tissue [67]. Physical therapy has been shown to provide some benefit in patients with Bell's palsy, with minimal risk to patients [4]. Chiropractic treatment has been shown to be successful in limited case studies [68]. Anatomically speaking, the upper cervical adjustment could have changed tension from the meninges, thus reducing a possible entrapment neuropathy [39]. There remains limited evidence to support chiropractic spinal manipulation for Bell’s palsy [2]. One study compared simple acupuncture therapy versus manipulative acupuncture therapy, which revealed that manipulative acupuncture had a significant recovery rate in the treatment of severe Bell's palsy and required a shorter course of treatment [69]. Botulinum toxin injection has been shown to be effective in restoring facial symmetry and reducing hyperkinesis, synkinesis, and facial imbalance due to facial palsy [70]. Another exciting new therapy is low-level laser therapy (LLLT) [8]. LLLT plus physiotherapy was reported to yield significant improvements when compared with physiotherapy alone [8]. One study showed that the recovery rate showed that LLLT is a safe, reliable, and proper alternative approach for the treatment of facial nerve palsy, especially in the presence of underlying conditions such as diabetes mellitus [71].

## Conclusions

It is well known that Bell’s palsy is a diagnosis of exclusion. Diagnosis should include ruling out a central lesion. The etiology and mechanism of Bell’s palsy and its deficits have been the subject of fierce debate for many, many years. It is shown that there are suspected comorbidities associated with the onset of Bell’s palsy such as obesity, diabetes, and pregnancy. While the neurological deficits are noticeable and at times debilitating, most patients will recover within six months regardless of the treatment.
